# Pluripotency of Dental Pulp Cells and Periodontal Ligament Cells Was Enhanced through Cell-Cell Communication via STAT3/Oct-4/Sox2 Signaling

**DOI:** 10.1155/2021/8898506

**Published:** 2021-01-19

**Authors:** Zhengjun Peng, Lu Liu, Wenyu Zhang, Xi Wei

**Affiliations:** Operative Dentistry and Endodontics, Guanghua School of Stomatology, Affiliated Stomatological Hospital, Guangdong Province Key Laboratory of Stomatology, Sun Yat-sen University, Guangzhou, Guangdong, China

## Abstract

Alternation in culture environment due to cell-cell communications can rejuvenate the biological activity of aged/differentiated cells and stimulate the expression of pluripotency markers. Dental pulp cells (DPCs) and periodontal ligament cells (PDLCs) are promising candidates in dental tissue regeneration. However, the molecular network that underlies cell-cell communications between dental-derived cells and the microenvironment remains to be identified. To elucidate the signaling network that regulates the pluripotency of DPCs and PDLCs, proliferation, apoptosis, cell cycle, and the expression of Oct-4/Sox2/c-Myc in DPCs and PDLCs with indirect/direct coculture were examined. PCR arrays were constructed to identify genes that were differentially expressed, and the results were confirmed by a rat model with injury. Further research on the mechanism of the related signaling pathways was investigated by overexpression/silence of STAT3, ChIP, the dual-luciferase reporter assay, and EMSA. We found that the proliferation and apoptosis of DPCs and PDLCs were inhibited, and their cell cycles were arrested at the G0/G1 phase after coculture. *Oct-4*, *Sox2*, and *STAT3* expression significantly increased and *PAX5* expression decreased in the coculture systems. Oct-4/Sox2/STAT3/PAX5 was actively expressed in the rat defect model. Moreover, STAT3 was directly bound to the *Oct-4* and *Sox2* gene promoter regions and activated the expression of those genes. Our data showed that the pluripotency of DPCs and PDLCs was enhanced through cell-cell communication. STAT3 plays essential roles in regulating the pluripotency of DPCs and PDLCs by targeting *Oct-4* and *Sox2* both *in vitro* and *in vivo.*

## 1. Introduction

Dental-derived cells are promising candidates for use in regenerating dental tissue. Dental pulp cells (DPCs) and periodontal ligament cells (PDLCs) can easily be obtained from dental tissues by using noninvasive procedures during a standard dental treatment. It has been demonstrated that DPCs and PDLCs can differentiate into odontogenic/osteogenic cells, adipocytes, and chondrogenic cells [1–4]. However, current *in vitro* culture methods might cause a loss of pluripotency and a decrease in the expression of pluripotent markers (Oct4, Sox2, and Stro1) in DPCs and PDLCs at later passages [5–8].

It has been reported that differentiated ESCs or iPSCs treated with resveratrol regain a naïve pluripotency state and express higher levels of core transcription factors. The treated cells can also differentiate to form all three germ layers by enhancing activation the JAK/STAT3 signaling pathway [9]. It is also known that a change in the culture environment, such as the addition of growth factors, can rejuvenate the biological activity of aged/differentiated cells and stimulate the expression of pluripotency markers [10, 11]. Coculture of DPCs with endothelial cells was shown to enhance the osteogenic/odontogenic properties of DPCs [12]. Therefore, there is growing interest in the signaling pathways involved in the regulation of cell-cell communications.

In our previous studies, we mimicked the *in vitro* tooth development model to investigate the expression of pluripotency factors Oct-4 and Sox2 in dental papilla and follicle cells with cell-cell interaction. Our results showed that the characteristics of dental papilla and follicle cells were modulated by the extrinsic environment [13]. In the present study, we established *in vitro* indirect and direct coculture systems to explore the specific signaling pathway and exact genes that regulate the pluripotency of DPCs and PDLCs with cell-cell interaction. The data presented in this report will help investigators understand how to increase the pluripotency of DPCs and PDLCs for their use in tissue engineering and dental regeneration.

## 2. Materials and Methods

### 2.1. Culture of DPCs and PDLCs

The protocol for this study was approved by the Ethics Committee of Sun Yat-sen University. DPCs and PDLCs were obtained from molars extracted from young human subjects (12-30 years old) during orthodontic treatment and then maintained in an explant culture as previously described [14, 15]. The third passages of DPCs and PDLCs were used in the subsequent experiments.

### 2.2. Lentivirus Transfection of Green Fluorescent Protein (GFP) into DPCs and PDLCs

The green fluorescent protein (GFP) gene was amplified from a plasmid and cloned into a lentivector. Plasmids of the recombinant gene and a lentivirus helper were cotransfected into HEK293T cells, which were then propagated. Lentivector carrying the *GFP* gene was used in the subsequent experiment. GFP expression in third passage DPCs and PDLCs was observed by a fluorescence microscope (Axiovert, Zeiss, Germany) at 48 h after transfection. The efficiency of viral transfer in the bulk population was estimated by flow cytometry (FACSCalibur; Becton Dickinson, Franklin Lakes, NJ, USA).

### 2.3. Heterochronic Pellet Assay

DPCs and PDLCs were prepared in the direct coculture system as previously described [16]. Briefly, DPCs (GFP+) (10^4^ cells/well), PDLCs (10^4^ cells/well) incubated for 1 h in BrdU, and DPCs (GFP+) plus PDLCs (BrdU+) (10^4^ cells/well) mixed thoroughly were seeded into tissue culture plates with slides, respectively. Replace half of media every second day. For immunostaining, DPCs (GFP+) and PDLCs (BrdU+) on the slides were harvested on days 3, 5, and 7. These cells were then fixed and successively pretreated and incubated with a 1 : 50 diluted primary antibody of BrdU (Abcam), secondary antibody (1 : 500 dilution), and DAPI (1 : 5000 dilution). Take images of DPCs (GFP+) and PDLCs (BrdU+) aggregates with an upright fluorescence microscope. Each group of experiments should be repeated at least three times.

### 2.4. Establishment of Cell-Cell Indirect and Direct Coculture Systems

The indirect coculture interaction model was established as previously described [13]. Briefly, DPCs and PDLCs were seeded into tissue culture plates (4 × 10^3^ cells/well) and cultured for 24 h. The PDLCs and DPCs were then seeded in the upper chambers and vice versa. The same numbers of cells were seeded into the bottom chambers to serve as the control groups.

In the direct coculture system, DPCs (GFP+) plus PDLCs and PDLCs (GFP+) plus DPCs were seeded into tissue culture plates at a density of 1 × 10^4^ cells/well and cultured for 24 h. The direct coculture cells and indirect coculture cells were maintained under identical conditions.

### 2.5. CCK8 Assays in the Indirect and Direct Coculture System

The proliferation rate of DPCs and PDLCs in the bottom wells was determined by CCK8 assays (Dojindo, Tokyo, Japan) that were performed according to the manufacturer's instructions. The proliferation of DPCs and PDLCs in the bottom wells on days 1 to 7 was evaluated using the CCK8 assay with the absorbance being measured at 450 nm.

DPCs and PDLCs with GFP in the direct coculture system were selected by flow cytometry. CCK8 assays (Dojindo, Tokyo, Japan) were also performed to evaluate the proliferation rate of DPCs and PDLCs with GFP according to the manufacturer's instructions.

### 2.6. Cell Cycle and Apoptosis Analysis in Both Systems

The cell cycle and apoptosis of DPCs and PDLCs were examined by flow cytometry. For cell cycle analysis, 1 × 10^5^ DPCs and PDLCs in the lower chambers were harvested on days 3 through 7. Next, single-cell suspensions were prepared, washed, and then incubated with propidium iodinate; after which, the cells were analyzed by flow cytometry. For apoptosis analysis, DPCs and PDLCs in the lower chambers were harvested on days 3 to 7, washed, and then resuspended in a mixture of AnnexinV-FITC and PI (MultiSciences Biotech, Shanghai, China) buffer in the dark. The cells were then analyzed by flow cytometry. Cells that were Annexin V+/PI- or Annexin V+/PI+ were considered to be apoptotic cells. DPCs (GFP+) and PDLCs (GFP+) with direct coculture were collected by fluorescence-activated cell sorting (FACS). The cell cycle and cell apoptosis analyses were performed as described above.

### 2.7. RT-PCR for *Oct-4*, *Sox2*, and *c-Myc* Expression

TRIzol reagent (Invitrogen, Carlsbad, CA, USA) was used to detect the expression of Oct-4, Sox2, and c-Myc mRNA in DPCs and PDLCs in the lower chambers on days 3, 5, and 7 according to the manufacturer's instructions. The levels of *Oct-4*, *Sox2*, *c-Myc*, and *GAPDH* gene expression were detected as previously described [13]. GAPDH served as a control housekeeping gene. The same methods were used to investigate the levels of *Oct-4*, *Sox2*, and *c-Myc* gene expression in the direct coculture system on days 3, 5, and 7.

### 2.8. Expression of Oct-4, Sox2, and c-Myc Proteins in DPCs and PDLCs in the Direct Coculture System

Western blotting was used to determine the levels of Oct-4, Sox2, and c-Myc protein expression in DPCs and PDLCs in the direct coculture system. Briefly, DPCs (GFP+) and PDLCs (GFP+) at passage 3 were harvested, lysed, sonicated, and then centrifuged. Next, a 50 *μ*g sample of total protein from each sample was separated by electrophoresis, and the protein bands were transferred onto nitrocellulose membranes, which were subsequently blocked. The membranes were then incubated with primary antibodies against Oct-4 (1 : 100, Proteintech, Chicago, IL, USA), Sox2 (1 : 50, Abcam, Cambridge, MA, USA), c-Myc (1 : 50, Epitomics, Burlingame, CA, USA), and GAPDH (1 : 1000, Santa Cruz Biotechnology, Dallas, TX, USA); after which, they were washed and incubated with a secondary antibody (1 : 5000, Jackson Laboratory, Bar Harbor, ME, USA). The immunostained protein bands were detected by enhanced chemiluminescence (GE Healthcare, Chicago, IL, USA).

### 2.9. Differential Expression of Stem Genes in the Indirect Coculture Cells

TRIzol reagent (Invitrogen) was used to extract the total RNA from DPCs and PDLCs in the lower chambers after 3 passages. Differentially expressed genes were detected by using stem cell-related RT2 Profiler PCR arrays (Super Array Bioscience Corporation, Frederick, MD, USA) according to the manufacturer's instructions. All procedures were performed using previously described methods [6]. Differentially expressed genes were defined as those which showed at least a 3-fold change in expression with a *P* value < 0.05 when compared with the control groups. The differentially expressed genes were classified into functional families according to their biological functions. The differential expression of stem genes was confirmed by western blotting.

### 2.10. Animal Model and Immunohistochemical Analysis

Lewis rats served as a source of dental pulp and models of periodontal ligament tissue injury, as described in a previous study [17]. All experimental procedures involving animals were the same as those used in our previous study [6] and were conducted according to guidelines established by the Sun Yat-sen University Ethics Committee. Briefly, the mandibles of rats in the surgery and control groups were removed and immediately separated between the central incisors. The mandibles were fixed in 4% paraformaldehyde, transferred into 5% methanoic acid, and then dehydrated using a gradient alcohol series. Following dehydration, the mandibles were paraffin embedded and cut into 5 *μ*m thick sections. The streptavidin-biotin-peroxidase complex technique was used to identify stem genes that were differentially expressed. Sections of tissue were successively pretreated and then incubated with the following antibodies: 1 : 100 dilution of Oct-4 (Proteintech), 1 : 50 dilution of Sox2 (Abcam), 1 : 100 dilution of STAT3 (Epitomics), and 1 : 100 dilution of PAX5 (Epitomics). Next, the tissue sections were washed and then incubated with a 1 : 200 diluted secondary antibody (Epitomics). In the negative control group, the primary antibody was replaced by PBS. The sections were counterstained with hematoxylin and then examined under an Olympus BX50 microscope (Tokyo, Japan).

### 2.11. Cell Transfection and Cell Biology Characteristics

pcDNA3.0-STAT3 and shRNA plasmid from human STAT3 were purchased from GenePharma in Guangzhou, China. For overexpression and silence, cells were plated in 24-well plates at a density of 10^4^/ml. After 24 h, cells were transfected with pcDNA3.0-STAT3 plasmid or shRNA plasmid using Lipofectamine 3000 (Life Technologies, MD, USA) following the manufacturer's instructions. The mRNA and protein levels were assessed 48 h later. TRIzol reagent (Invitrogen, Carlsbad, CA, USA) was used to detect the expression of *STAT3*, *Oct-4*, and *Sox2* mRNA in DPCs and PDLCs according to the manufacturer's instructions. Western blotting was used to determine the levels of STAT3, Oct-4, and Sox2 protein expression in DPCs and PDLCs. Apoptosis and cell cycle of DPCs and PDLCs were examined by flow cytometry.

### 2.12. Dual-Luciferase Reporter Assay and Transfections

The target genes for transcription factor STAT3 were identified by TargetScan. pGL3 plasmids (Promega, Madison, WI, USA) containing a STAT3 binding site for wild-type (WT) or MUT (mutated STAT3 binding site) Oct-4/Sox2 were amplified and then transfected along with STAT3 mimics into HEK293T cells. A control vector (pTK-RL) containing the Renilla luciferase gene was added to the transfection mix. Transfections were performed by using Lipofectamine 2000 (Invitrogen) according to the manufacturer's protocol. After 48 h of transfection, luciferase (Firefly/Renilla) activity was measured using the Dual-Glo™ Luciferase Assay System (Promega) as described in the manufacturer's protocol.

### 2.13. Chromatin Immunoprecipitation (ChIP)

The chromatin immunoprecipitation (ChIP) assay was used to investigate the targeting relationship of the immune complexes as described in the manufacturer's protocol. Cross-linked chromatin was obtained from DPCs in the lower chambers after they had been treated with formaldehyde and sodium dodecyl sulfate. Next, the chromatin was incubated with anti-STAT3 rabbit polyclonal antibody (Cell Signaling Technology, Danvers, MA, USA) or normal rabbit IgG (Cell Signaling Technology) overnight at 4°C. The precipitated DNA was purified and analyzed by qRT-PCR. The primers used for detection are listed in Table 1.

### 2.14. Electrophoretic Mobility Shift Assay (EMSA)

The mechanism by which STAT3 regulates Oct-4/Sox2 expression was verified by the electrophoretic mobility shift assay (EMSA). In brief, two double-stranded oligonucleotide probes identical to STAT3 in the Oct-4 and Sox2 promoter regions were synthesized and labeled with Digoxigenin-11-ddUTP (DIG-ddUTP). The probes used for investigation are listed in Table 1. DNA-protein complexes derived from the nuclei of DPCs in the lower chambers were assayed with labeled probes in the presence or absence of specific/mutant competitors or an anti-STAT3 antibody. Samples of the binding products were separated by electrophoresis and then visualized by autoradiography according to instructions provided with a 2nd generation DIG Gel Shift Kit (Roche, Mannheim, Germany).

### 2.15. Statistical Analysis

All data were analyzed using IBM SPSS Statistics for Windows, Version 19.0 software (IBM Corp., Armonk, NY, USA), and results are presented as the mean value ± standard deviation (SD). Differences between cells in the coculture and control groups were analyzed by the Student's *t*-test. A *P* value < 0.05 was considered to be statistically significant.

## 3. Results

### 3.1. Expression of GFP in DPCs and PDLCs

**Figure 1:**
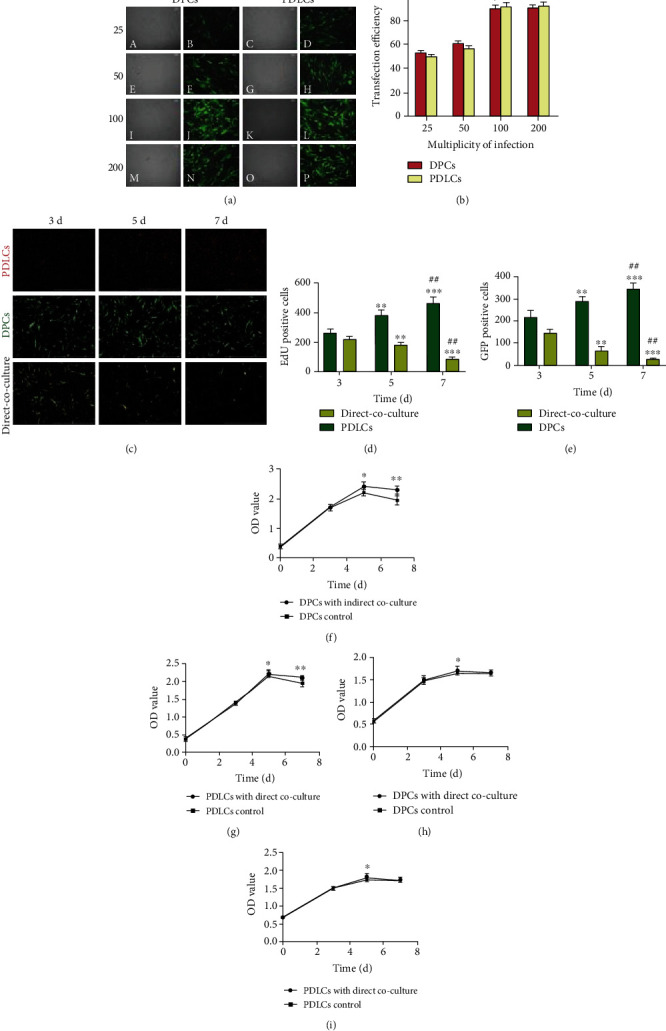
Expression of GFP in PDCs and PDLCs, and the proliferation rate of PDCs and PDLCs. GFP was visually detected in lentivirus-transduced clones after transduction (a). The efficiency of lentivirus transduction was 89.3% ± 1.5% in DPCs and 91.3% ± 1.9% in PDLCs at a multiplicity of infection (MOI) of 100 (b). Representative images of DPCs and PDLCs were taken with upright fluorescence microscope at different time points (c). DPCs (GFP+), PDLCs (BrdU+), and DPCs (GFP+) plus PDLCs (BrdU+) were seeded and incubated into tissue culture plates for 3, 5, and 7 d, respectively. Data was presented in the histogram (d, e). The proliferation rates of cocultured DPCs (GFP+) and PDLCs (BrdU+) were significantly reduced (^∗∗^*P* < 0.01, ^∗∗∗^*P* < 0.001, and ^##^*P* < 0.01). The proliferation rate of DPCs and PDLCs with indirect or direct coculture was significantly reduced on day 5 as compared with control cells (f, g, h, and i, ^∗^*P* < 0.05 and ^∗∗^*P* < 0.01). Scale bars = 100 *μ*m.

The lentivirus-transduced clones were found to be stably transduced. After transduction, GFP was detected under a fluorescence microscope (Figure 1(a)). The efficiency of viral transfer was calculated by determining the proportion of GFP-expressing cells with a FACSCalibur flow cytometer. The efficiency of lentivirus transduction was 89.3% ± 1.5% in DPCs and 91.3% ± 1.9% in PDLCs at a multiplicity of infection (MOI) of 100, and the efficiency increased in proportion to the MOI (Figure 1(b)). There was no statistically significant difference in the efficiency of transduction at MOIs of 100 and 200.

### 3.2. Heterochronic Cocultures of DPCs and PDLCs

We repeated three times and took ten photomicrographs each group to count the cell numbers of DPCs (GFP+) and PDLCs (BrdU+). Parts of the results are shown (Figure 1(c)). The ratio of proliferation of DPCs (GFP+) and PDLCs (BrdU+) is calculated with the following formula: %DPCs (GFP+)^5 d^/DPCs (GFP+)^3 d^ and %PDLCs (BrdU+)^5 d^/PDLCs (BrdU+)^3 d^. Data was presented in the histogram (Figures 1(d) and 1(e)). The proliferation rates of cocultured DPCs (GFP+) and PDLCs (BrdU+) were significantly reduced compared with the control groups (^∗∗^*P* < 0.01, ^∗∗∗^*P* < 0.001, and ^##^*P* < 0.01).

### 3.3. The Proliferation, Cell Cycle, and Apoptosis of DPCs and PDLCs

**Figure 2:**
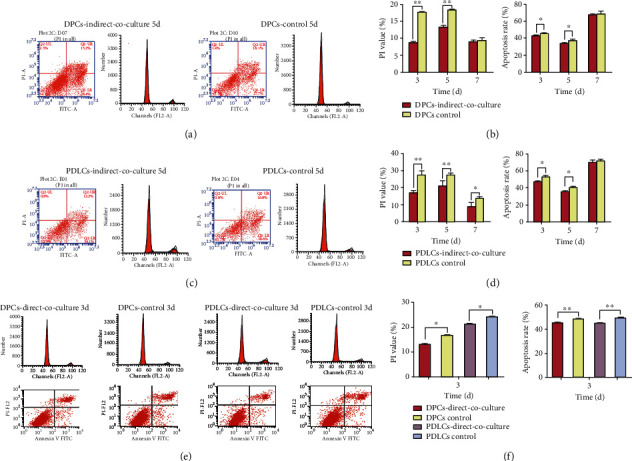
Cell cycle and apoptosis of DPCs and PDLCs with coculture. (a–d) The cell cycle phases of DPCs and PDLCs were arrested at the G0/G1 phase on 3 and 5 days of indirect coculture. The percentage of propidium iodinate value (PI) = (S + G2/M)% in cells with indirect coculture was significantly lower than those in the control groups (^∗^*P* < 0.05 and ^∗∗^*P* < 0.01). The apoptosis rates of DPCs and PDLCs were significantly downregulated on 3 and 5 days of indirect coculture (^∗^*P* < 0.05 and ^∗∗^*P* < 0.01). (a, c) Part of the results of cell cycle and apoptosis of DPCs and PDLCs performed by flow cytometry on 5 days of indirect coculture was represented, respectively. (e, f) The cell cycle phases and apoptosis rates of DPCs and PDLCs with 3 days of direct coculture are presented. The percentage of propidium iodinate value (PI) = (S + G2/M)% in cells with direct coculture was significantly lower than those in the control groups (^∗^*P* < 0.05 and ^∗∗^*P* < 0.01). The cell apoptosis rates of DPCs and PDLCs were significantly downregulated on 3 days of direct coculture (f, Table 3, ^∗^*P* < 0.05 and ^∗∗^*P* < 0.01). (e) Part of the results of cell cycle and apoptosis of DPCs and PDLCs performed by flow cytometry on days 3 of direct coculture was represented.

Changes in cell proliferation, the cell cycle, and apoptosis of DPCs and PDLCs with 3, 5, and 7 days of *in vitro* indirect and direct coculture were investigated. Cell cycle and apoptosis performed were represented, respectively (Figures 2(a), 2(c), and 2(e)). The proliferation rates of DPCs and PDLCs were significantly reduced on days 5 of indirect or direct coculture (Figures 1(f)–1(i), ^∗^*P* < 0.05 and ^∗∗^*P* < 0.01). The cell cycles of both DPCs and PDLCs with indirect coculture were arrested at the G0/G1 phase on days 3 and 5. Notably, the percentage of propidium iodinate value (PI) = (S + G2/M)% in cells with indirect coculture was significantly lower than that in the control groups. The apoptosis rates of DPCs and PDLCs were significantly reduced on days 3 and 5 of indirect coculture (Table 2 and Figures 2(b) and 2(d), ^∗^*P* < 0.05 and ^∗∗^*P* < 0.01).

The percentage of propidium iodinate value (PI) = (S + G2/M)% in DPCs and PDLCs with 3 days of direct coculture was significantly lower than that in the control groups. The apoptosis rates of DPCs and PDLCs were significantly reduced after 3 days of direct coculture (Figure 2(f) and Table 3, ^∗^*P* < 0.05 and ^∗∗^*P* < 0.01). Thus, the coculture system appeared to reduce cell proliferation, force cells to remain in their G0/G1 phase, and inhibit cell apoptosis. Therefore, both coculture systems (indirect and direct) might force DPCs and PDLCs into a quiescent state by preventing cell proliferation and lowering the PI value.

### 3.4. The mRNA Expression for *Oct-4*, *Sox2*, and *c-Myc* in DPCs and PDLCs with Coculture

**Figure 3:**
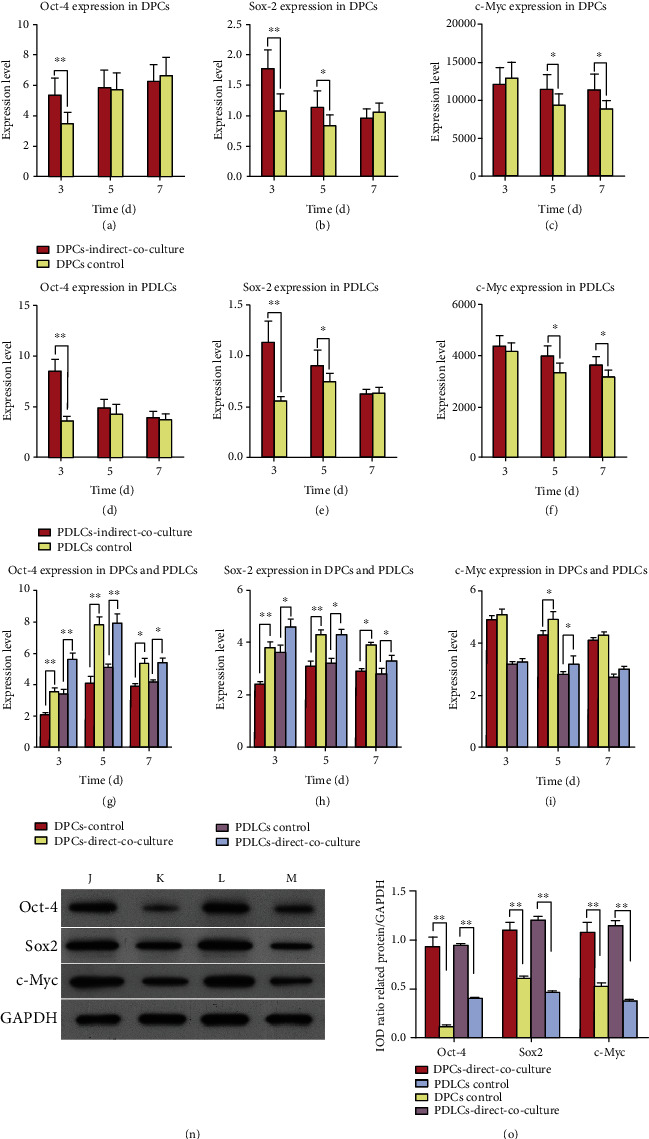
The levels of Oct-4, Sox2, and c-Myc mRNA and protein expression in DPCs and PDLCs with coculture. The levels of Oct-4 (a, d) and Sox2 (b, e) mRNA in DPCs and PDLCs were significantly upregulated on 3 days of indirect coculture when compared with those levels in the control groups (^∗∗^*P* < 0.01). c-Myc (c, f) expression showed no significant change in DPCs and PDLCs with indirect coculture until day 5 (^∗^*P* < 0.05). Oct-4 (g) and Sox2 (h) showed similar patterns of expression in DPCs and PDLCs with direct coculture and were significantly upregulated on days 3 and 5 (^∗^*P* < 0.05 and ^∗∗^*P* < 0.01). c-Myc (i) expression showed no significant change in DPCs and PDLCs with direct coculture until day 5 (^∗^*P* < 0.05). The expression of Oct-4, Sox2, and c-Myc proteins was strongly enhanced in DPCs and PDLCs with direct coculture (n, o). (j) DPCs with direct coculture. (k) DPCs control. (l) PDLCs with direct coculture. (m) PDLCs control.

In the indirect coculture system, *Oct-4* (Figures 3(a) and 3(d)) and *Sox2* (Figures 3(b) and 3(e)) showed similar patterns of mRNA expression in both DPCs and PDLCs, and both mRNA levels were significantly upregulated on day 3 (^∗∗^*P* < 0.01). *c-Myc* (Figures 3(c) and 3(f)) mRNA expression showed no significant change in DPCs and PDLCs until day 5 (^∗^*P* < 0.05).

In the direct coculture system, *Oct-4* (Figure 3(g)) and *Sox2* (Figure 3(h)) mRNA expressions were significantly upregulated on days 3, 5, and 7 compared with the control groups (^∗^*P* < 0.05 and ^∗∗^*P* < 0.01). *c-Myc* (Figure 3(i)) mRNA expression showed no significant change in DPCs and PDLCs on days 3 and 7 except day 5 (^∗^*P* < 0.05).

### 3.5. Expression of Pluripotency Protein Markers in DPCs and PDLCs with Direct Coculture

The levels of Oct-4, Sox2, and c-Myc protein expression were confirmed by western blot studies, and representative data from passage 3 cells are shown in Figure 3. The results showed that the levels of Oct-4, Sox2, and c-Myc proteins were significantly increased in DPCs and PDLCs with direct coculture (Figures 3(o) and 3(p)), which was in agreement with our real-time PCR results. This suggests that Oct-4 and Sox2 might function cooperatively to regulate downstream markers and maintain the pluripotency of DPCs and PDLCs via cell-cell interaction.

### 3.6. Gene Expression Profiles in DPCs and PDLCs with Indirect Coculture

**Figure 4:**
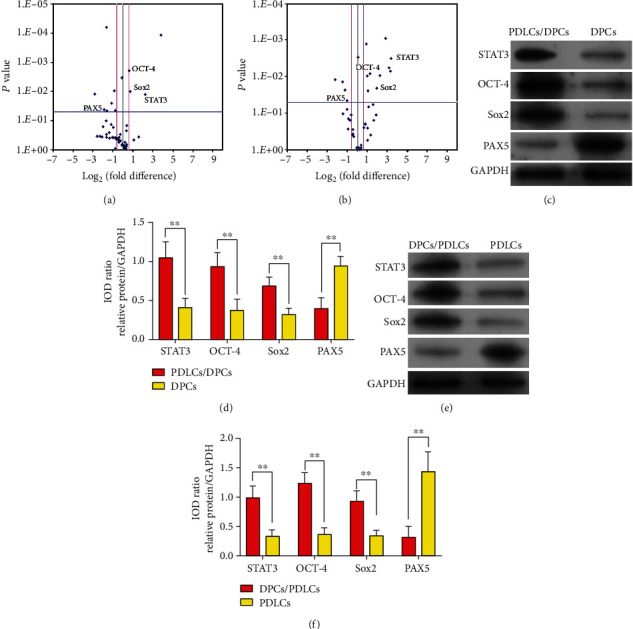
Differentially expressed genes in DPCs and PDLCs with indirect coculture. The *x*-axis (a log transformation plot of the fold difference) and the *y*-axis (*P* value) indicate the differential expression of each gene in the indirect coculture and control groups of DPCs (a) and PDLCs (b). The pink lines indicate a 3-fold up- or downregulation of gene expression. The blue line indicates the threshold for the *P* value (*P* < 0.05) of the *t*-test. In the indirect coculture systems, four genes (*ZFPM2*, *STAT3*, *SOX2*, and *OCT-4*) were significantly upregulated, and seven genes (*EGR3*, *PAX5*, *PCNA*, *STAT1*, *RUNX1*, *FGF1*, and *NOTCH2*) were significantly downregulated in the indirect cocultured DPCs. Meanwhile, ten genes (*STAT3*, *HOXC10*, *HOXA9*, *EZH2*, *ESR1*, *SOX2*, *OCT-4*, *DLX2*, *PPARG*, and *KLF4*) were significantly upregulated, whereas four genes (*PAX1*, *NANOG*, *HOXA7*, and *PAX5*) were significantly downregulated in the indirect cocultured PDLCs. Western blot analyses showed that the levels of STAT3, OCT-4, and SOX2 protein expression were strongly upregulated and PAX5 expression was downregulated in DPCs (c, d) and PDLCs (e, f) after indirect coculture.

The PCR array system was used to assess the expression of genes in DPCs and PDLCs undergoing indirect coculture. The *x*-axis (log transformation plots of the fold difference) and the *y*-axis (*P* value) of differentially expressed genes in the coculture and control groups are shown in Figures 4(a) and 4(b). Four genes (*ZFPM2*, *STAT3*, *SOX2*, and *OCT-4*) showed significantly increased expression, and seven genes (*EGR3*, *PAX5*, *PCNA*, *STAT1*, *RUNX1*, *FGF1*, and *NOTCH2*) showed significantly decreased expression in DPCs with indirect coculture (Figure 4 and Table 4). Ten genes (*STAT3*, *HOXC10*, *HOXA9*, *EZH2*, *ESR1*, *SOX2*, *OCT-4*, *DLX2*, *PPARG*, and *KLF4*) showed significantly increased expression, and four genes (*PAX1*, *NANOG*, *HOXA7*, and *PAX5*) showed significantly decreased expression in PDLCs with indirect coculture (Figure 4 and Table 5).

Interestingly, three genes were overexpressed (*STAT3*, *OCT-4*, and *SOX2*), while barely one gene was underexpressed (*PAX5*) in DPCs and PDLCs with indirect coculture. Western blot studies (Figures 4(c) and 4(e)) demonstrated that the levels of STAT3, OCT-4, and SOX2 protein expression were significantly increased, while the PAX5 protein was only slightly expressed in DPCs and PDLCs after indirect coculture, which agreed with our PCR array results.

### 3.7. Expression of Stem Cell-Related Markers in the Rat Injury Model

**Figure 5:**
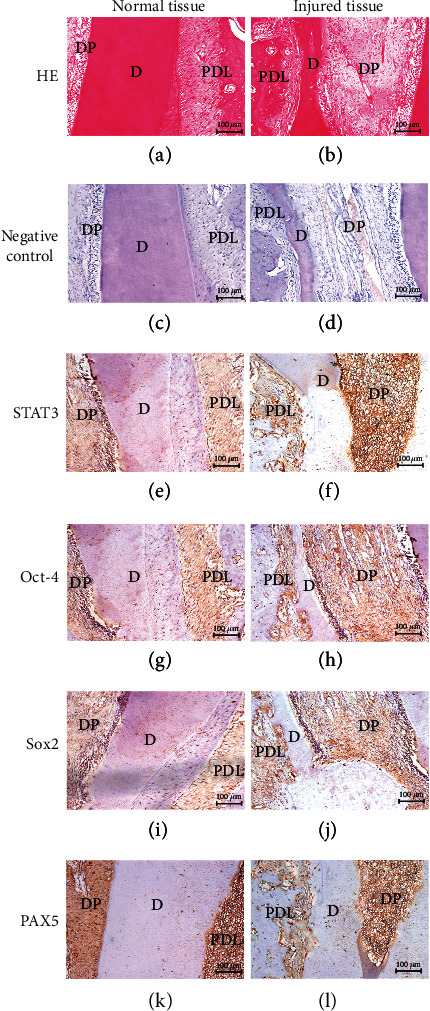
Expression and distribution of stem cell-related markers in the rat defect models. Hematoxylin and eosin staining revealed the structures of the normal and injury groups (a, b). The primary antibody was replaced by PBS in the negative control groups of normal and injury groups (c, d). STAT3, Oct-4, and Sox2 were notably present in the nucleus and, to a lesser extent, in the cytoplasm of the injury groups (f, h, and j). Rare staining of STAT3, Oct-4, and Sox2 was found in the normal groups (e, g, and i). Intense staining of PAX5 was found in the cytoplasm and matrix around the normal dental pulp and periodontal ligament when compared with PAX5 staining in the repaired connective tissues (k). Rare staining of PAX5 was detected in the injury groups (l). Scale bars = 100 *μ*m. D: dentin; DP: dental pulp; PDL: periodontal ligament.

The normal and wound healing areas of rat dental tissue were stained with hematoxylin and eosin (Figures 5(a) and 5(b)). The expression of stem cell-related markers STAT3, Oct-4, Sox2, and PAX5 was confirmed by immunohistochemical staining. The levels of STAT3 (Figures 5(e) and 5(f)), Oct-4 (Figures 5(g) and 5(h)), and Sox2 (Figures 5(i) and 5(j)) protein expression in the injury model rats were significantly increased when compared with those in the normal dental pulp/periodontal tissues. Moreover, those proteins were mainly expressed in the nucleus of the cells, with some moderate expression being located in the cytoplasm of DPCs, PDLCs, and odontoblasts. In contrast, the PAX5 protein (Figures 5(k) and 5(l)) was much more strongly expressed in the normal tissues than in the injury model. In the negative control group, the primary antibody was replaced by PBS (Figures 5(c) and 5(d)). The distribution of STAT3, Oct-4, Sox2, and PAX5 proteins was detected in the regenerated pulp-dentin complex tissues and found to be mostly limited to the cell nucleus (Figures 5(e), 5(g), 5(i), and 5(k)).

### 3.8. Cell Biology Characteristics in DPCs and PDLCs with Overexpression or Silence of STAT3

**Figure 6:**
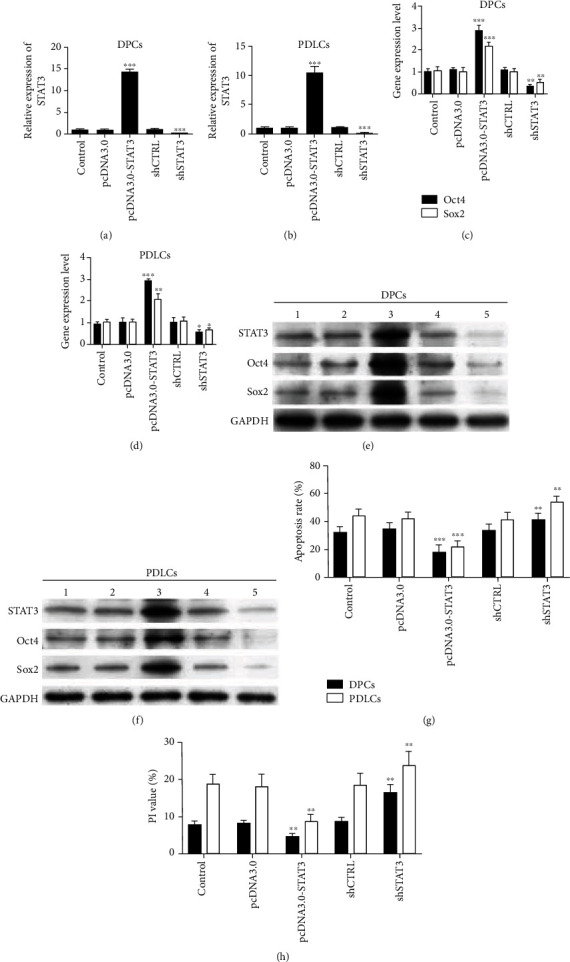
STAT3 overexpression and silence models were established in DPCs and PDLCs. The results of PCR and WB showed that STAT3 was significantly enhanced and silenced in DPCs and PDLCs (a, b, e, and f, ^∗∗∗^*P* < 0.001). *Oct-4* (c, d) and *Sox2* (c, d) showed similar patterns of mRNA expression, consistent with the overexpression or silence of *STAT3*, and both mRNA levels were significantly upregulated or downregulated in both DPCs and PDLCs (^∗^*P* < 0.05, ^∗∗^*P* < 0.01, and ^∗∗∗^*P* < 0.001). The results of western blot showed that the levels of Oct-4 and Sox2 proteins were significantly increased or reduced in DPCs and PDLCs (e, f). The apoptosis rates of DPCs and PDLCs were significantly reduced compared with the control group (g, Table 6, ^∗∗^*P* < 0.01 and ^∗∗∗^*P* < 0.001). The cell cycles of both DPCs and PDLCs were arrested at the G0/G1 phase (h, Table 6, ^∗∗^*P* < 0.01). 1: group of control; 2: group of pcDNA3.0; 3: group of pcDNA3.0-STAT3; 4: group of shCTRL; and 5: group of shSTAT3.

System of overexpression and silence of STAT3 in DPCs and PDLCs was established. The results of PCR and WB indicated that STAT3 was significantly enhanced and silenced in DPCs and PDLCs (Figures 6(a), 6(b), 6(e), and 6(f), ^∗∗∗^*P* < 0.001). *Oct-4* and *Sox2* showed similar patterns of mRNA expression, consistent with the overexpression or silence of *STAT3*, which significantly upregulated with overexpression of *STAT3* or downregulated with silence of *STAT3* in both cells (Figures 6(c) and 6(d), ^∗^*P* < 0.05, ^∗∗^*P* < 0.01, and ^∗∗∗^*P* < 0.001). Similarly, western blot demonstrated that Oct-4 and Sox2 proteins were significantly increased with overexpression of STAT3 or reduced with silence of STAT3 (Figures 6(e) and 6(f)). Apoptosis rates of DPCs and PDLCs were significantly reduced compared with the control group (Figure 6(g) and Table 6, ^∗∗^*P* < 0.01). Cell cycles of both DPCs and PDLCs were arrested at the G0/G1 phase (Figure 6(h) and Table 6, ^∗∗^*P* < 0.01).

### 3.9. Targeting Relationship between STAT3 and Oct-4/Sox2

**Figure 7:**
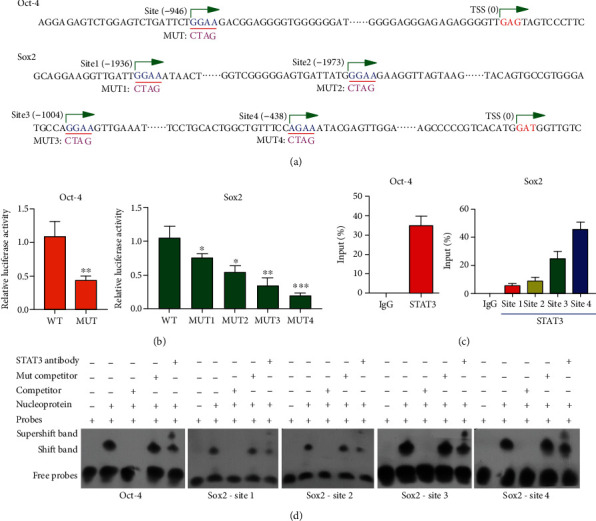
STAT3 bound to the endogenous Oct-4/Sox2 promoters. (a) Oligonucleotide sequences indicating the Oct-4/Sox2 promoters and the distance from the transcription start site (TSS) are shown. Green arrows denote the transcription direction. (b) The relative luciferase activities of the Oct-4 and Sox2 mutants were significantly decreased when compared with the STAT3 mimic in cotransfected 293 T cells. Data are represented as the mean ± SD. ^∗^*P* < 0.05, ^∗∗^*P* < 0.01, and ^∗∗∗^*P* < 0.001; wild-type (WT) vs. mutation (MUT). (c) STAT3 binding to the endogenous Oct-4 and Sox2 promoters was evaluated by the chromatin immunoprecipitation assay. The levels of Oct-4 and Sox2 promoter regions in the lower chamber DPCs were significantly higher than those in the control group. (d) The binding sites for STAT3 on *Oct 4* and *Sox2* were detected by the electrophoretic mobility shift assay (EMSA) performed with DIG-labeled oligonucleotide probes (lane 2). Incubation with a specific competitor abolished the binding (lane 3), while addition of a mutant competitor increased the intensities of the putative bands (lane 4). Moreover, incubation with STAT3 antibody reduced the intensities of those bands while causing an upward super shift (lane 5).

The TargetScan online tool was used to investigate the target gene of STAT3. *Oct-4* with one binding region and *Sox2* with four binding regions were identified as possible targets of STAT3 (Figure 7(a)). The luciferase activities of the *Oct-4* mutant and *Sox2* mutant were significantly lower than those of the STAT3 mimic in 293 T cells (Figure 7(b), ^∗^*P* < 0.05, ^∗∗^*P* < 0.01, and ^∗∗∗^*P* < 0.001). These results indicated that *Oct-4* and *Sox2* were targets of STAT3.

ChIP assays were performed with DPCs in the lower chambers to verify whether STAT3 binds to the endogenous *Oct-4* and *Sox2* promoter regions (Figure 7(c)). Consistent with our previous findings, the Oct-4 and Sox2 promoter regions were present at significantly higher levels than in the control group.

Oligonucleotide probes with mutations in the Oct-4/Sox2 motif sites were synthesized, and EMSAs were performed (Figure 7(d)). The binding sites for STAT3 on the Oct-4 promoter and Sox2 promoters 1, 2, 3, and 4 were detected (lane 2). Incubation with a specific competitor abolished the binding (lane 3), while addition of a mutant competitor increased the intensity of the putative bands (lane 4). Moreover, incubation with an anti-STAT3 antibody reduced the intensity of those bands, and an upward super shift was observed (lane 5). These results indicated that *Oct-4* and *Sox2* were the target genes for STAT3 in DPCs, and STAT3 directly correlates with Oct-4/Sox2.

## 4. Discussion

Dental pulp has a regenerative ability due to the presence of DPCs that contain progenitor stem cells [14, 18]. These cells are recruited and participate in the process of dentin regeneration via odontoblast-like cell differentiation [19]. PDLCs with a specific phenotypic profile represent a valuable reservoir of autologous stem cells that are able to ensure dental regeneration. It was reported that the coculturing of endothelial cells with DPCs could enhance the odontogenic properties of the DPCs and also promote the formation blood vessel-like structures formed by endothelial cells [12]. Nevertheless, the mechanisms of interactions between extrinsic signals and intrinsically acting factors remain unknown. With the present coculture system, the quantity of upregulated genes in PDLCs was far more than that in DPCs. This suggested that compared with DPCs, more genes might be involved in maintaining the states of pluripotency and nonmineralization of PDLCs. A reasonable explanation of this result might be that PDLCs have the ability to maintain the balance needed to form cementum and bone, and keep themselves in a nonmineralized status. Since cell characteristics can be greatly affected by the culture environment, the specific signals that regulate cell-cell communication need to be identified.

In this study, cells became arrested in their G0/G1 phase, and their rate of apoptosis became downregulated after 3 and 5 days of coculture. Meanwhile, the levels of Oct-4, Sox2, and c-Myc expression were clearly upregulated after 3 and 5 days of coculture. These results were in high agreement with our previous study [13]. An earlier report included a detailed discussion of the mechanistic links between reprogramming, pluripotency, and the cell cycle. *Oct-4* and *Sox2* were shown to control the cell cycle through miRNA expression. The Myc family was shown to comprise well-defined regulators of cell cycle progression and be able to impact Cdk activity to regulate cell size and drive cells into their S-phase [20]. Our results indicated that the apoptosis rate showed a similar expression trend with proliferation in DPCs and PDLCs with direct or indirect coculture. However, these findings regarding relationships between reprogramming, pluripotency, and the cell cycle were somewhat unexpected and require further clarification. Another report suggested that reducing somatic cell proliferation could increase the generation of induced pluripotent stem cells [21].

Our previous study showed that the levels of Oct-4, Sox2, and c-Myc expression in dental papilla and follicle cells became enhanced both *in vivo* and *in vitro* after direct or indirect cell-cell contact. Similarly, our present data revealed that the expressions of stem-related markers STAT3, Oct-4, Sox2, and c-Myc were enhanced in both DPCs and PDLCs by cell-cell interaction. Notably, three genes were overexpressed (*STAT3*, *Oct-4*, and *Sox2*), whereas barely one gene (*PAX5*) was underexpressed in DPCs and PDLCs with indirect coculture. This suggests that STAT3, Oct-4, and Sox2 might play essential roles in maintaining the pluripotency of DPCs and PDLCs by promoting cell-cell communication.

Oct-4, a member of the POU5 family, is a master regulator of gene transcription in pluripotent cells and is vital for the formation of initial pluripotent cell populations. This is because Oct-4 promotes the transcription of stem cell-specific genes and inhibits the transcription of tissue-specific genes. Oct-4 is necessary to maintain cell self-renewal, and its up/downregulation triggers divergent developmental processes [22]. Sox2, a member of the HMG box transcription factor family, plays a key role in precursor cells of the early embryo and their *in vitro* equivalents [23]. Sox2 is involved in cell self-renewal and differentiation [24]. Unlike Oct-4 and Sox2, there is no reliable evidence to support Myc involvement in the maintenance of cell pluripotency. However, previous studies have suggested that increased Myc activity leads to many changes in the cycle characteristics of pluripotent cells. Once the Myc miRNA function is lost, p21 and cyclin D2 expression become upregulated, which results in the loss of pluripotency [25, 26]. Myc appears to be critical for maintaining a proper balance of cell cycle regulatory molecules involved in a tightly interconnected mechanism that regulates cell pluripotency. In conjunction with Oct-4, Sox2, and Klf4, c-Myc regulates ESC-specific miRNA expression that could be used as an alternative to Myc in the process of cell reprogramming [27], and plays a role in enhancing global histone acetylation in order to silence genes associated with cell differentiation [28]. Our current results revealed that the levels of Oct-4 and Sox2 were significantly elevated in both DPCs and PDLCs after coculture, indicating that Oct-4 and Sox2 might cooperatively serve to maintain pluripotency by regulating the downstream markers.

Signal transducer and activator of transcription 3 (STAT3) was first discovered as the downstream factor of interleukin 6 (IL-6) and was shown to have multiple functions in regulating cell proliferation, pluripotency, immune responses, and differentiation [29]. Enhanced STAT3 activity was shown to promote a complete reprogramming process in OKSM-induced partially reprogrammed cells, neural stem cells, and mouse epiblast stem cells (mEpiSCs) [9]. STAT3 activation can also promote the reprogramming of somatic stem cells by regulating certain essential factors, such as the upstream factors *Smad7* and *Esrrb* and downstream target, *Gbx2* [30–32]. Activated STAT3 is imported into the nuclei to bind with tissue-specific response factors in different cells, such as *Grg5* in ES cells [33] and *Bcl3* in B cells [34]. Those factors are then able to regulate the expression of certain downstream genes, such as *Oct-4* and *Nanog* [35]. Some studies concluded that STAT3 promotes the reprogramming process mainly via epigenetic regulation. One study demonstrated that inhibition of LIF/STAT3 directly affected the demethylation of *Oct-4* and *Nanog* enhancer-promoter regions by downregulating DNA methyltransferase 1 (Dnmt 1) expression [36]. An earlier study indicated STAT3, Oct-4, and Sox2 suppressed the expression of differentiation-related genes by cobinding to the promoter region of the *Eed* gene [37]. Recent studies demonstrated that phosphorylated STAT3, with amino acid sequences for recognizing and binding to specific DNA sequences, was imported into the nuclei or mitochondria to regulate the expression of downstream genes [35, 38]. Activated STAT3 was shown to increase the expression levels of mitochondrial genes through direct binding of the STAT3 protein onto mtDNA, and thereby alter ATP production to maintain cell pluripotency. However, activated STAT3 also directly targets the core regulatory network genes *Tfcp211*, *Klf4*, and *Gbx2* to maintain naive pluripotency [32, 39–41]. Previous studies demonstrated that the efficiency of reprogramming was abolished or reduced even if Oct-4 and Sox2 were over-expressed while STAT3 activity was inhibited [9, 42]. In this study, the levels STAT3, Oct-4, and Sox2 were significantly elevated in both DPCs and PDLCs after 3 days of coculture. In order to elucidate the regulating roles STAT3 and Oct-4/Sox2 played, STAT3 overexpression and silence models were established in DPCs and PDLCs. Based on the similar expression patterns of STAT3/Oct-4/Sox2, we speculated that the biological behaviors of DPCs and PDLCs, such as apoptosis and pluripotency, might be regulated by the STAT3/Oct-4/Sox2 signaling pathway. Further mechanistic research indicated that activated STAT3 enters the cell nucleus to directly target the *Oct-4*/*Sox2* promoters, and then controls the expression of its target genes *Oct-4*/*Sox2*.

PAX5 was originally shown to potentially regulate *CD19* gene expression [43]. PAX5 could induce the expression of c-Myc, which participates in regulating the cell cycle, cell proliferation, apoptosis, and differentiation. While the normal expression of c-Myc is exquisitely regulated, abnormally high levels of c-Myc expression cause the activation of key downstream genes, and eventually DNA replication and cell cycle progression [44–46]. An earlier study indicated that pro-B cells lacking PAX5 were incapable of differentiation *in vitro* unless Pax5 expression was restored by retroviral transduction [42]. In this study, we found that DPCs and PDLCs maintained their pluripotent capacity and showed low rates of proliferation and apoptosis after coculture, which might be closely related to a low level of PAX5 expression.

Both *in vivo* and *in vitro*, microenvironment interacts with cells to regulate the proliferation, differentiation and angiogenesis, and cell fate determination [47, 48]. Studies have shown that coculture of dental pulp stem cells and vascular endothelial cells can promote bone formation and angiogenesis [49]. Oct-4 and Sox2 are pluripotency markers, seldomly expressed in differentiated tissues, herein activated expressed in injured dental tissues. It might be possible that the pluripotency network was activated in DPCs and PDLCs after injury through cell-cell communication, and the dental-derived cells could migrate to the injured site and initiate the tissue regeneration process. The present study provides a novel insight to the potential application of dental-derived cells to dental tissue regeneration. Nevertheless, further studies would be required to elucidate the specific signaling regulation of the regeneration process in the future.

## 5. Conclusions

In summary, we demonstrated that the pluripotency of DPCs and PDLCs was enhanced via cell-cell communication. STAT3 plays essential roles in regulating the pluripotency of DPCs and PDLCs by targeting *Oct-4/Sox2* both *in vitro* and *in vivo.* Our findings suggest new strategies for improving pluripotency and can assist in identifying the key signals that regulate the pluripotency of dental-derived cells.

## Figures and Tables

**Table 1:** Primers and probes used in ChIP and EMSA.

Gene	Primers and probes
Oct-4	Forward: 5′-CCTTGAAGGGGAAGTAGGAC-3′
	Reverse: 5′-CAAGGCCTCCGTGCTATATCC-3′
	Probe: 5′-TGGAGTCTGATTCTGGAAGACGGAGGGGTGGGG-3′

Sox2 site 1	Forward: 5′-CTTGAGAGAAAAAGGAGAAC-3′
	Reverse: 5′-CACACTAAATATACCCACTGG-3′
	Probe: 5′-GCAGGAAGGTTGATTGGAAATAACTTAAGGAA-3′

Sox2 site 2	Forward: 5′-GCAGAGATTGGAGAAATTGG-3′
	Reverse: 5′-GTATCTACCAGCCACGTTCC-3′
	Probe: 5′-TCGGGGGAGTGATTATGGGAAGAAGGTTAGTAA-3′

Sox2 site 3	Forward: 5′-GGGAGGGAGTTTGTGACTGC-3′
	Reverse: 5′-GGCGCTCAAAAGTGCAGGCG-3′
	Probe: 5′-GTGCCGTGGGATGCCAGGAAGTTGAAATCACCC-3′

Sox2 site 4	Forward: 5′-GGAGTGCTGTGGATGAGCGG-3′
	Reverse: 5′-GGATGGGACGCGGGAAGCAG-3′
	Probe: 5′-CTGGCTGTTTCCAGAAATACGAGTTGGACAGCC-3′

**Table 2:** The cell cycle and apoptosis of DPCs and PDLCs with indirect coculture (mean ± SD%; *N* = 3).

Time point of culture	G0/G1 (%)	PI(S + G2/M)%	Apoptosis(FITC+/PI±)%
DPCs-indirect coculture 3 d	48.3 ± 0.4^∗^	8.8 ± 0.2^∗∗^	42.9 ± 0.4^∗^
DPCs-control 3 d	44.7 ± 0.3	17.3 ± 0.5	46.7 ± 0.2
DPCs-indirect coculture 5 d	46.2 ± 0.5^∗^	13.7 ± 0.4^∗∗^	36.4 ± 0.3^∗^
DPCs-control 5 d	43.4 ± 0.4	18.6 ± 0.3	38.5 ± 0.4
DPCs-indirect coculture 7 d	45.8 ± 0.2	9.8 ± 0.4	67.2 ± 0.6
DPCs-control 7 d	45.4 ± 0.2	10.1 ± 0.3	69.5 ± 0.3
PDLCs-indirect coculture 3 d	46.3 ± 0.3^∗^	18.2 ± 0.2^∗∗^	48.5 ± 0.4^∗^
PDLCs-control 3 d	41.1 ± 0.4	26.4 ± 0.4	52.4 ± 0.5
PDLCs-indirect coculture 5 d	45.5 ± 0.3^∗^	22.2 ± 0.4^∗∗^	36.7 ± 0.4^∗^
PDLCs-control 5 d	42.1 ± 0.4	25.3 ± 0.2	39.8 ± 0.5
PDLCs-indirect coculture 7 d	46.1 ± 0.3^∗^	7.8 ± 0.3^∗^	68.5 ± 0.3
PDLCs-control 7 d	42.4 ± 0.5	13.5 ± 0.2	70.4 ± 0.2

Significant upregulation of the cell percentage was observed in DPCs and PDLCs in the G0/G1 phase after 3 d and 5 d of coculture, with a corresponding decrease of cell apoptosis. The significant decrease of cell cycle in the G0/G1 phase was maintained in PDLCs at 7 d of indirect coculture (^∗∗^*P* < 0.01 and ^∗^*P* < 0.05). However, there is no statistical significance of cell cycle in the G0/G1 phase in DPCs and PDLCs after 7 days of indirect coculture (*P* > 0.05). DPCs-indirect coculture: DPCs located in lower chamber with PDLCs in upper chamber; DPCs-control: DPCs located in lower chamber without PDLCs; PDLCs-indirect coculture: PDLCs located in lower chamber with DPCs in upper chamber; PDLCs-control: PDLCs located in lower chamber without DPCs; PI: propidium iodinate (percentage of cells in S + G2/M phases); apoptosis: FITC+/PI- (early apoptotic) or FITC+/PI+ (late apoptotic).

**Table 3:** The cell cycle and apoptosis of PDCs and PDLCs with direct coculture at 3 d (mean ± SD%; *N* = 3).

Day	G0/G1	PI = (S + G2/M)%	(FITC+/PI±)%
DPCs-direct coculture	43.8 ± 0.3^∗^	13.2 ± 0.2^∗∗^	45.3 ± 0.4^∗^
DPCs-control	40.1 ± 0.4	16.7 ± 0.4	48.5 ± 0.3
PDLCs-direct coculture	45.6 ± 0.3^∗^	21.3 ± 0.2^∗∗^	44.9 ± 0.4^∗^
PDLCs-control	40.2 ± 0.4	24.2 ± 0.3	49.3 ± 0.5

Significant upregulation was observed in the percentage of direct cocultured DPCs and PDLCs in the G0/G1 phase at day 3 of direct coculture, with a corresponding decrease of cell apoptosis (^∗∗^*P* < 0.01 and ^∗^*P* < 0.05). PI: propidium iodinate (percentage of cells in S + G2/M phases); apoptosis: FITC+/PI- (early apoptotic) or FITC+/PI+ (late apoptotic).

**Table 4:** List of genes with altered expression with DPCs with indirect coculture.

Gene name	Gene symbol	Fold difference (PDLCs/DPCs vs. DPCs)	*P* value	Functional gene grouping
Zinc finger protein, multitype 2	ZFPM2	13.77	0.0401	Embryonic development Organ morphogenesis

Signal transducer and activator of transcription 3 (acute-phase response factor)	STAT3	8.64	0.0129	Induced pluripotent and embryonic stem cell Organ morphogenesis Neurogenesis

SRY- (sex determining region Y-) box 2	SOX2	3.65	0.0180	Somatic stem cell maintenance Induced pluripotent and embryonic stem cell Embryonic development Organ morphogenesis Neurogenesis Osteogenesis

Octamer-binding transcription factor 4	OCT-4	3.53	0.0020	Somatic stem cell maintenance Induced pluripotent and embryonic stem cell

Early growth response 3	EGR3	-8.84	0.0164	Oncogenesis

Paired box 5	PAX5	-7.42	0.0137	Organ morphogenesis

Proliferating cell nuclear antigen	PCNA	-6.66	0.0452	Cell cycle DNA replication

Signal transducer and activator of transcription 1, 91 kDa	STAT1	-6.05	<0.0001	Hematopoiesis Proliferation

Runt-related transcription factor 1	RUNX1	-5.16	0.0158	Organ morphogenesis Angiogenesis Hematopoiesis

Fibroblast growth factor 1	FGF 1	-4.01	0.0459	Cell cycle regulator Cytokines and growth factor Endoderm and mesoderm formation and differentiation Organ morphogenesis

Notch 2	NOTCH2	-3.26	0.0097	Segmentation/axis/symmetry Embryonic development Organ morphogenesis

**Table 5:** List of genes with altered expression with PDLCs with indirect coculture.

Gene name	Gene symbol	Fold difference (DPCs/PDLCs vs. PDLCs)	*P* value	Functional gene grouping
Signal transducer and activator of transcription 3 (acute-phase response factor)	STAT3	10.06	0.0031	Induced pluripotent and embryonic stem cell Organ morphogenesis Neurogenesis

Homeobox C10	HOXC10	9.66	0.0069	Segmentation/axis/symmetry Embryonic development Neurogenesis

Homeobox A9	HOXA9	8.84	0.0057	Segmentation/axis/symmetry Embryonic development

Enhancer of zeste homolog 2 (Drosophila)	EZH2	6.92	0.0009	Cell growth Resistance to apoptosis

Estrogen receptor 1	ESR1	4.66	0.0093	Oncogenesis

SRY- (sex determining region Y-) box 2	SOX2	4.36	0.0024	Somatic stem cell maintenance Induced pluripotent and embryonic stem cell Embryonic development Organ morphogenesis Neurogenesis Osteogenesis

Octamer-binding transcription factor 4	OCT-4	3.85	0.0082	Somatic stem cell maintenance Induced pluripotent and embryonic stem cell

Distal-less homeobox 2	DLX2	3.25	0.0442	Embryonic development Organ morphogenesis Neurogenesis

Peroxisome proliferator-activated receptor gamma	PPARG	3.14	0.0012	Placenta development Organ morphogenesis Neurogenesis

Kruppel-like factor 4 (gut)	KLF4	3.03	0.0095	Embryonic development Ectoderm, endoderm, and mesoderm formation and differentiation Organ morphogenesis

Paired box 1	PAX1	-7.32	0.0121	Organ morphogenesis

Nanog homeobox	NANOG	-5.21	0.0139	Somatic stem cell maintenance Induced pluripotent and embryonic stem cell Embryonic development

Homeobox A 7	HOXA7	-4.75	0.0204	Segmentation/axis/symmetry Embryonic development Ectoderm, endoderm, and mesoderm formation and differentiation

Paired box 5	PAX5	-4.39	0.0232	Organ morphogenesis

**Table 6:** Apoptosis and cell cycle of PDCs and PDLCs in overexpression and silence models (mean ± SD%; *N* = 3).

Group	Apoptosis (%)	PI (%)
DPCs-control	36.8 ± 0.3	8.2 ± 0.2
DPCs-pcDNA 3.0	37.6 ± 0.4	8.4 ± 0.1
DPCs-pcDNA 3.0-STAT3	18.3 ± 0.3^∗∗∗^	4.8 ± 0.2^∗∗^
DPCs-shCTRL	37.2 ± 0.4	8.5 ± 0.1
DPCs-shSTAT3	40.8 ± 0.2^∗∗^	16.3 ± 0.2^∗∗^
PDLCs-control	43.1 ± 0.2	18.7 ± 0.1
PDLCs-pcDNA 3.0	41.2 ± 0.3	17.3 ± 0.2
PDLCs-pcDNA 3.0-STAT3	22.2 ± 0.1^∗∗∗^	8.4 ± 0.1^∗∗^
PDLCs-shCTRL	39.8 ± 0.4	17.1 ± 0.2
PDLCs-shSTAT3	52.1 ± 0.2^∗∗^	23.1 ± 0.1^∗∗^

The apoptosis rates of DPCs and PDLCs were significantly reduced compared with the control group. The cell cycles of both DPCs and PDLCs were arrested at the G0/G1 phase (^∗∗^*P* < 0.01 and ^∗∗∗^*P* < 0.001). PI: propidium iodinate (percentage of cells in S + G2/M phases).

## Data Availability

The data included in this study are available upon request by contact with the corresponding author.
